# Reduction of N-acetyl aspartate (NAA) in association with relapse in early-stage psychosis: a 7-Tesla MRS study

**DOI:** 10.1038/s41537-024-00451-7

**Published:** 2024-03-01

**Authors:** Marina Mihaljevic, Yu-Ho Chang, Ashley M. Witmer, Jennifer M. Coughlin, David J. Schretlen, Peter B. Barker, Kun Yang, Akira Sawa

**Affiliations:** 1 Departments of Neuroscience, Johns Hopkins University School of Medicine, Baltimore, MD USA; 2 Department of Mental Health, Johns Hopkins Bloomberg School of Public Health, Baltimore, MD USA; 3 Departments of Psychiatry, Johns Hopkins University School of Medicine, Baltimore, MD USA; 4 Departments of Radiology, Johns Hopkins University School of Medicine, Baltimore, MD USA; 5 Departments of Biomedical Engineering, Johns Hopkins University School of Medicine, Baltimore, MD USA; 6 Departments of Genetic Medicine, Johns Hopkins University School of Medicine, Baltimore, MD USA; 7 Departments of Pharmacology, Johns Hopkins University School of Medicine, Baltimore, MD USA

**Keywords:** Molecular neuroscience, Psychosis

## Abstract

Understanding the biological underpinning of relapse could improve the outcomes of patients with psychosis. Relapse is elicited by multiple reasons/triggers, but the consequence frequently accompanies deteriorations of brain function, leading to poor prognosis. Structural brain imaging studies have recently been pioneered to address this question, but a lack of molecular investigations is a knowledge gap. Following a criterion used for recent publications by others, we defined the experiences of relapse by hospitalization(s) due to psychotic exacerbation. We hypothesized that relapse-associated molecules might be underscored from the neurometabolites whose levels have been different between overall patients with early-stage psychosis and healthy subjects in our previous report. In the present study, we observed a significant decrease in the levels of N-acetyl aspartate in the anterior cingulate cortex and thalamus in patients who experienced relapse compared to patients who did not. Altogether, decreased N-acetyl aspartate levels may indicate relapse-associated deterioration of neuronal networks in patients.

## Introduction

The outcomes following the first episode of psychosis (FEP) exhibit significant heterogeneity in terms of symptom severity, patient functionality, and treatment response^1^. Among the various contributors to heterogeneous outcomes after FEP, relapse emerges as one of the main features of poor disease trajectory^2^. Relapse is highly frequent in psychosis, affecting approximately 30–50% of patients within the first three years following disease onset^3^. As a result, combating relapse has become a major goal in the management and treatment of psychosis.

There are two complementary lines of effort in addressing relapse. First, there are efforts to identify risk factors and triggers that elicit relapse, which include treatment non-adherence, substance abuse, and stressful life events^3^. One of the major breakthroughs in this line of efforts was a recent report of the dose-dependent relationship between stressful life events and the incidence of relapse, in which hospitalization was used as a measure for relapse^4^. Second, there are efforts that address the possible impact of relapse as a negative consequence. One good example is a trajectory study based on the staging model of psychosis^5^. Although the reasons for relapse can vary, current literature suggests that brain alterations may occur as a consequence of relapse^6–8^. While these studies are intriguing, most of them predominantly focused on investigating brain connectivity or structural changes, with a lack of molecular investigations.

To address this knowledge gap, we conducted a study building upon our previous publication on a 7-Tesla (T) magnetic resonance spectroscopy (MRS) study, which highlighted significant differences in patients in the early stages of psychosis (within 2 years of onset) compared to healthy controls^9^. Specifically, our previous study indicated that psychotic patients had lower levels of glutamate (Glu), γ-aminobutyric acid (GABA), N-acetylaspartate (NAA), and glutathione (GSH) in the anterior cingulate cortex (ACC), as well as reduced NAA levels in the orbitofrontal cortex (OFR) and thalamus, and diminished GSH levels in the thalamus^9^. We hypothesize that relapse may lead to alterations of the brain, which are represented as changes in brain metabolites. NAA and GSH directly reflect neuron health and dysfunction^10^, which may possibly lead to changes in neurotransmitters such as Glu and GABA. Altogether, we further hypothesize that patients who experience relapse may show more significant changes in these metabolites compared with those who do not.

## Methods

Participants were scanned in a 7 T scanner (Philips ‘Achieva’, Best, The Netherlands) equipped with a 32-channel head coil (Nova Medical, Wilmington, MA) for this MRS study. As published^9,11,12^, the ‘LCModel’ software package (Version 6.3-0D) was used to analyze the spectra. Metabolite levels relative to total creatine (tCr) or water signal were calculated from the same voxel and expressed in institutional units (IU, approximately millimolar).

Patients with a DSM-IV diagnosis of psychotic disorder (see details in the Supplementary Information) within two years of the onset were recruited for this study. We did not enroll patients who reported active substance abuse. Furthermore, we excluded the patients who were positive in a urine screen test for illicit substance use, with the exception of cannabis use, following the standards used in other psychosis studies^13,14^.

We collected demographic data (age, sex, race), clinical data (diagnosis, duration of illness, antipsychotic dosage at the time of MRS scan, cannabis use), and neuropsychological scores. The antipsychotic dosages were converted to chlorpromazine equivalents (CPZ) using the defined daily dose method^15^, and the duration of illness (DOI) represented the time between onset and the MRS scan.

We used a comprehensive neuropsychological battery to obtain cognitive scores that covered six domains: processing speed, attention/working memory, verbal learning and memory, visual learning and memory, ideational fluency, and executive functioning^12^. Additional details are available in our previous publications^9,11,12^.

This study was approved by the Johns Hopkins Medicine Institutional Review Boards and in accordance with The Code of Ethics of the World Medical Association (1964 Declaration of Helsinki). All study participants provided written informed consent. Parental consent and assent were obtained for all participants under the age of 18 years.

We stratified the patients into two subgroups based on the hospitalization records between the onset of psychosis and the MRS scan: patients who had hospitalization(s) due to psychotic exacerbation after the onset were assigned to the relapsed (R) group, while the remaining patients without hospitalization were assigned to the non-relapsed (NR) group. Recent studies showed that re-admission into hospitals (re-hospitalization) is a frequently used and reliable measure of relapse^4,16^. In addition, we carefully examined medical records for treatment adherence, duration between hospitalizations, and cannabis use to determine recovery or symptom stability prior to psychotic symptom exacerbation. Patients who were nonadherent to medication and/or used substances frequently prior to hospitalizations were excluded from this study. In total, data from 24 patients in the R group and 38 patients in the NR group were used.

Analysis of covariance (ANCOVA) with age, sex, race, diagnosis (see details in the Supplementary Information), cannabis use, CPZ dose, and DOI as covariates was conducted to compare MRS data between the R and NR groups. General linear regression with the same covariates was employed to test the association between neuropsychological scores and neurometabolites (only significant neurometabolites identified by group comparison between the R and NR groups were tested). The Benjamini and Hochberg procedure was used for multiple comparison correction. More details about statistical analysis are available in the Supplementary Information.

## Results

In demographic and clinical data between the R and NR groups, no significant differences were observed in demographic data between the groups, while the clinical data indicated a longer duration of illness and higher antipsychotic dosage in the R group compared with the NR group (Table S1). Also, there was no significant difference in LC model quality metrics (full width at half-maximum, signal-to-noise ratios, Cramér-Rao lower bounds), white matter, gray matter, cerebrospinal fluid (CSF), and tCr in the ACC, thalamus, and OFR between the R and NR groups (Table S2).

**Fig. 1:**
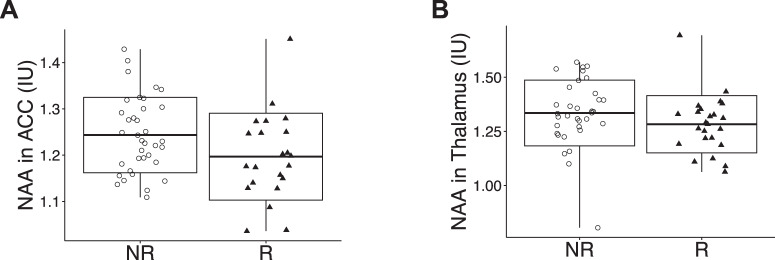
Boxplots of significant neurometabolites between R and NR groups. **A** NAA levels in the thalamus. **B** NAA levels in the ACC.

We next compared the levels of NAA, Glu, GSH, and GABA in the ACC, NAA and GSH in the thalamus, as well as NAA in the OFR, between the R and NR groups. After multiple comparison corrections, our analysis of MRS data normalized by the tCr signal revealed that the R group exhibited significantly decreased NAA levels in the ACC and thalamus compared to the NR group (Fig. 1), while no significant differences were found in other neurometabolites (Table S3). When we used the water signal for normalization, no significant differences were observed after multiple comparison corrections (Table S3). We further assessed whether the differences observed in NAA levels in the ACC and thalamus between R and NR groups may be associated with CPZ dose and DOI. Accordingly, we tested the correlations between the ACC NAA and CPZ, the ACC NAA and DOI, the thalamus NAA and CPZ, and the thalamus NAA and DOI in patients. No significant correlations were observed (Table S4), indicating that the lower levels of NAA are likely to be associated with relapse itself rather than other confounding factors.

**Fig. 2:**
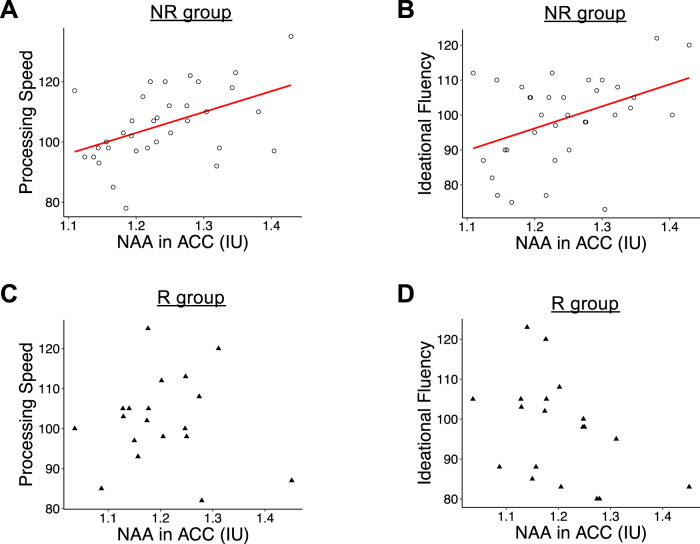
Dot plots of associations between NAA levels (relative to tCr) in the ACC region and neuropsychological scores. **A** Processing speed in the NR group; **B** ideational fluency in the NR group; **C** processing speed in the R group; **D** ideational fluency in the R group.

A recent meta-analysis showed that decreased NAA level could be the strongest marker of progression from mild cognitive impairment to Alzheimer’s disease^17^. Therefore, we tested associations between NAA levels in the ACC and thalamus (normalized by tCr) and neuropsychological scores. NAA levels in the ACC were positively correlated with processing speed and ideational fluency in the NR group, but not in the R group (Fig. 2, Table S5).

## Discussion

This study represents the first investigation of relapse-associated brain metabolite alterations in early-stage psychosis. Studying the consequence of relapse may also help interpret longitudinal changes observed in psychotic patients during disease progression^11,18^. We found significantly decreased NAA levels in the ACC and thalamus in the R group compared to the NR group. In addition, we found that higher NAA levels in the ACC were correlated with better neuropsychological functions (processing speed and ideational fluency) in the NR group, but such positive correlations were lost in the R group.

NAA is thought to reflect neuronal health and integrity^10^. Moreover, lower NAA in living patients may be reflected in the synaptic and mitochondrial deficits^19^. Consistent with these notions, a reduction of NAA has been reported in neurodegenerative disorders^20,21^. Although robust neurodegeneration is not observed in psychotic disorders, a mild but significant reduction of NAA has been reported in multiple reports^22,23^, including our past publication^9,11,12^. The significantly decreased NAA levels in the ACC and thalamus in patients with psychotic disorders who experienced relapse compared to those who did not imply that the biological consequence of relapse may accompany a functional deterioration in the neuronal network. This notion may amount to a poorer prognosis for psychosis patients who experience relapse compared with those who maintain remission^1,24,25^. The ACC has been frequently underscored in neuroimaging studies for psychotic diseases, including our previous research demonstrating its involvement in social cognition^26^ and poor prognosis in psychosis^12^. Furthermore, we have recently highlighted the pathological role of the thalamus in association with relapse in a study of resting-state functional connectivity^27^, which may have complementary implications to the molecular observations reported in the present study.

We observed that NAA levels in the ACC were significantly correlated with processing speed and ideational fluency in the NR group but seemed to be lost in the R group. Although we cannot draw any biological message for these molecular-neuropsychological relationships with the current sample size, we hope that this observation may aid future investigations on molecular changes in association with relapse.

The majority of the patients in this study were medicated. Thus, it is important to consider the potential effects of medications on the levels of NAA. As far as we look for the past literature, antipsychotics do not significantly affect the levels of brain NAA shown in longitudinal studies of psychotic patients^28,29^. Consistently, no effects of antipsychotics on the brain NAA were observed in animal study^30^. Furthermore, in vitro experiments with human SH-SY5Y neuroblastoma cells indicated that antipsychotics could potentially elevate NAA levels^31^. In the present study, we showed that the influence of relapse on ACC NAA levels exhibits a much larger effect size compared to the impact of CPZ dose (Fig. 1). Taken together, although this may be a point that we need to carefully and conservatively pay attention to, there is no definitive conclusion regarding the impact of medications on the brain NAA.

Here, we present results from data normalized by both the water and the total creatine (tCr) signal. We observed significant results in the data normalized by tCr, but not in the data normalized by water. Both the water signal and tCr are commonly used internal references in MRS studies; each has its own advantages and disadvantages^32^. Meta-analysis studies of diffusion magnetic resonance imaging (MRI) data found significant differences in extracellular free water signal between schizophrenia and controls^33,34^. In contrast, no significant alternations in tCr between schizophrenia and controls have been observed in meta-analysis studies^35,36^. We did not observe any significant difference in tCr between the two groups investigated in this study (R and NR groups) either. Nevertheless, at the individual paper level, there have been a small number of studies reporting significant alterations in tCr between schizophrenia and controls^37,38^. The MRS expert consensus paper^32^ published in 2022 discusses referencing to both tCr and water in detail but makes no recommendations as to which is better or more appropriate to use, particularly in pathological conditions where neither signal may be normal. Further exploration is warranted to understand how the choice of internal reference relates to both the significant and non-significant results observed in this study.

We acknowledge the limitations of the present study: these include no measurement of stressful life events and insufficient statistical power to address the effects of different types of medications. Future longitudinal studies with data before and after relapse, as well as complete records about the triggers of each relapse, could further help us evaluate the consequence of relapse and its independence (or dependence) from the triggers. In addition, patients with a longer duration of illness often experienced more number of relapses^3^. While there was no significant correlation between duration of illness and NAA levels in our cohort, future studies are encouraged to dissect the effects of duration of illness and cumulative impacts of relapse on the brain. Nevertheless, we believe that the present report of a potential association of lower NAA levels with relapse as its consequence may shed light on future mechanistic studies for relapse. We believe that intervening with brain changes based on mechanistic information, as this study demonstrated, in parallel to preventing relapse by identifying its risk factors and triggers, are complementary endeavors essential for enhancing the management of psychosis treatment.

### Supplementary information

Supplementary Information

## Data Availability

MRS. and clinical data are available to academic researchers upon request under Johns Hopkins Material Transfer Agreements.
