# Supplementary material for: Reduction of N-acetyl aspartate (NAA) in association with relapse in early-stage psychosis: a 7-Tesla MRS study
Source: Schizophrenia (Heidelb). 2024 Mar 1;10(1):29. doi: 10.1038/s41537-024-00451-7 (PMC10907360; doi:10.1038/s41537-024-00451-7)
Supplement: Supplementary file 1 — Supplementary Information [file 41537_2024_451_MOESM1_ESM.docx]

**Supplementary Information for**

**Reduction of N-acetyl aspartate (NAA) in association with relapse in early-stage psychosis: a 7- Tesla MRS study**

Marina Mihaljevic^1^, Yu-Ho Chang^7^, Ashley M. Witmer^7^, Jennifer M. Coughlin^2^, David J. Schretlen^2^, Peter B. Barker^3^, Kun Yang^2*^, and Akira Sawa^1,2,4,5,6,7*^

Departments of Neuroscience^1^, Psychiatry^2^, Radiology^3^, Biomedical Engineering^4^, and Genetic Medicine^5^, Pharmacology^6^, Johns Hopkins University School of Medicine, Baltimore, MD

Department of Mental Health^7^, Johns Hopkins Bloomberg School of Public Health, Baltimore, MD.

*Correspondence authors

Akira Sawa (contact): asawa1@jhmi.edu; Kun Yang: kunyang@jhmi.edu

Postal address: 600 N Wolfe ST, Baltimore, MD 21287, USA

**Study participants**

In this study, we utilized data from 62 psychotic patients, with the following DSM-IV diagnoses: schizophrenia (n = 33), schizoaffective disorder (n = 7), schizophreniform disorder (n = 2), bipolar disorder with psychosis (n = 11), major depressive disorder with psychotic features (n = 5), and not otherwise specified psychotic disorder (n=4).

**Statistical analysis**

The R 3.5.1 statistical software was used to perform the data analysis. T-test and chi-squared test were conducted to compare the demographical and clinical data between R and NR groups for continuous (age, chlorpromazine equivalents (CPZ), duration of illness (DOI)) and categorical (sex, race, cannabis use) variables, respectively. Two-sided analysis of covariance (ANCOVA) with age, sex, race, diagnosis, cannabis use (Yes/No), CPZ dose, and DOI as covariates was conducted to compare LC model quality metrics (full width at half-maximum, signal-to-noise ratios, Cramér-Rao lower bounds), white matter, gray matter, cerebrospinal fluid (CSF), and total creatine (tCr) in the anterior cingulate cortex (ACC), thalamus, and orbitofrontal cortex (OFR) between the R and NR groups.

We next employed ANCOVA with age, sex, race, diagnosis, cannabis use, CPZ dose, and DOI as covariates to compare MRS data between the R and NR groups. One-sided test was conducted to test whether the pathological changes of neurometabolites, observed in patients compared with healthy controls, were more profound in the R group compared with the NR group. To further assess whether the significant changes between the R and NR groups identified by ANCOVA may be associated with CPZ dose and DOI, we conducted two-sided Pearson correlation analysis to test the correlations between the significant neurometabolites and CPZ dose and DOI in patients.

Lastly, we employed a general linear model with age, sex, race, diagnosis, cannabis use, CPZ dose, and DOI as covariates to test the association between neuropsychological scores and neurometabolites. In this analysis, two-sided test was conducted and only significant neurometabolites identified by ANCOVA between the R and NR groups were tested. The Benjamini and Hochberg procedure was used for multiple comparison correction when multiple statistical tests were involved. Results with adjusted p-values (also called q-values) smaller than 0.05 were considered significant.

**Table S1. Differences in demographic and clinical characteristics between R and NR group**

|  | R  (n=24) | NR  (n=38) | R vs. NR  p-value |
| --- | --- | --- | --- |
| Age  (mean ± SD) | 22.9±3.9 | 22.7±4.9 | 0.844 |
| Sex  (male %) | 70.8 | 68.4 | 0.797 |
| Race  (white %) | 25.0 | 42.1 | 0.104 |
| Cannabis use  (yes %) | 37.5 | 23.7 | 0.379 |
| CPZ  (mean ± SD) | 364.7±222.9 | 232.4±188.6 | **0.033** |
| DOI  (months, mean ± SD) | 19.9±8.6 | 11.9±9.4 | **0.004** |

**Table S2. Group comparison results of LC model quality metrics, CSF, white matter, gray matter, and total creatine in the ACC, thalamus, and OFR between the R and NR groups**

**A. Analysis results of CSF, gray matter, white matter, full width at half-maximum (FWHM), signal-to-noise ratio (SNR), and total creatine**

| Metabolite | Brain region | mean (R) | SD (R) | mean (NR) | SD (NR) | p-value |
| --- | --- | --- | --- | --- | --- | --- |
| CSF | ACC | 0.010 | 0.032 | 0.008 | 0.027 | 0.883 |
| Gray matter | ACC | -0.017 | 0.020 | -0.007 | 0.018 | 0.403 |
| White matter | ACC | 0.007 | 0.016 | -0.001 | 0.016 | 0.482 |
| FWHM | ACC | 0.029 | 0.003 | 0.030 | 0.004 | 0.955 |
| SNR | ACC | 54.609 | 5.631 | 52.583 | 3.813 | 0.255 |
| Total creatine | ACC | 5.878 | 0.560 | 5.818 | 0.419 | 0.245 |
| CSF | Thalamus | 0.006 | 0.012 | 0.008 | 0.018 | 0.163 |
| Gray matter | Thalamus | 0.010 | 0.042 | -0.016 | 0.044 | 0.731 |
| White matter | Thalamus | -0.016 | 0.042 | 0.008 | 0.046 | 0.929 |
| FWHM | Thalamus | 0.050 | 0.014 | 0.048 | 0.010 | 0.175 |
| SNR | Thalamus | 18.217 | 6.809 | 17.639 | 7.695 | 0.452 |
| Total creatine | Thalamus | 5.883 | 0.753 | 5.816 | 0.615 | 0.112 |
| CSF | Orbitofrontal cortex | 0.000 | 0.007 | 0.000 | 0.002 | 0.956 |
| Gray matter | Orbitofrontal cortex | -0.048 | 0.076 | -0.005 | 0.044 | 0.357 |
| White matter | Orbitofrontal cortex | 0.048 | 0.075 | 0.005 | 0.045 | 0.358 |
| FWHM | Orbitofrontal cortex | 0.049 | 0.010 | 0.050 | 0.010 | 0.529 |
| SNR | Orbitofrontal cortex | 22.739 | 6.662 | 21.583 | 6.362 | 0.883 |
| Total creatine | Orbitofrontal cortex | 5.569 | 0.512 | 5.622 | 0.726 | 0.365 |

**B. Analysis results of Cramér-Rao lower bound (CRLB)**

| Metabolite | Brain region | mean (R) | SD (R) | mean (NR) | SD (NR) | p-value |
| --- | --- | --- | --- | --- | --- | --- |
| GSH | ACC | 4.125 | 0.338 | 4.316 | 0.525 | 0.083 |
| GABA | ACC | 5.542 | 0.658 | 5.737 | 0.724 | 0.097 |
| GSH | Thalamus | 10.000 | 3.375 | 11.200 | 5.465 | 0.475 |
| NAA | Orbitofrontal cortex | 2.500 | 0.511 | 2.842 | 1.263 | 0.605 |
| NAA | ACC | 1.917 | 0.282 | 1.974 | 0.162 | 0.677 |
| Glu | ACC | 1.792 | 0.415 | 1.921 | 0.273 | 0.791 |
| NAA | Thalamus | 3.167 | 0.868 | 3.421 | 1.348 | 0.883 |

**Table S3. Group comparison results of neurometabolites between R and NR group**

**A. Analysis results of MRS data normalized by the total creatine signal**

| Metabolite | Brain region | mean (R) | SD (R) | mean (NR) | SD (NR) | p-value | q-value* |
| --- | --- | --- | --- | --- | --- | --- | --- |
| **NAA** | **ACC** | **1.197** | **0.094** | **1.244** | **0.081** | **0.006** | **0.027** |
| **NAA** | **Thalamus** | **1.283** | **0.132** | **1.335** | **0.152** | **0.008** | **0.027** |
| Glu | ACC | 1.360 | 0.075 | 1.378 | 0.101 | 0.031 | 0.073 |
| GABA | ACC | 0.286 | 0.033 | 0.290 | 0.032 | 0.176 | 0.247 |
| GSH | ACC | 0.246 | 0.023 | 0.250 | 0.026 | 0.209 | 0.247 |
| NAA | Orbitofrontal cortex | 1.385 | 0.150 | 1.388 | 0.185 | 0.212 | 0.247 |
| GSH | Thalamus | 0.253 | 0.043 | 0.241 | 0.050 | 0.465 | 0.465 |

* Multiple comparison correction for 7 tests was conducted

**B. Analysis results of MRS data normalized by the water signal**

| Metabolite | Brain region | mean (R) | SD (R) | mean (NR) | SD (NR) | p-value | q-value* |
| --- | --- | --- | --- | --- | --- | --- | --- |
| NAA | ACC | 7.040 | 0.470 | 7.205 | 0.511 | 0.063 | 0.310 |
| NAA | Orbitofrontal cortex | 7.632 | 0.675 | 7.745 | 0.842 | 0.089 | 0.310 |
| GSH | Thalamus | 1.520 | 0.352 | 1.398 | 0.321 | 0.153 | 0.358 |
| Glu | ACC | 7.964 | 0.641 | 7.996 | 0.478 | 0.279 | 0.461 |
| NAA | Thalamus | 7.466 | 0.858 | 7.564 | 0.845 | 0.353 | 0.461 |
| GABA | ACC | 1.672 | 0.181 | 1.691 | 0.154 | 0.395 | 0.461 |
| GSH | ACC | 1.451 | 0.157 | 1.463 | 0.121 | 0.482 | 0.482 |

* Multiple comparison correction for 7 tests was conducted

**Table S4. The correlation between NAA levels and clinical variables**

| Metabolite | Brain region | clinical variable | p-value |
| --- | --- | --- | --- |
| NAA | ACC | DOI | 0.193 |
| NAA | ACC | CPZ dose | 0.507 |
| NAA | Thalamus | DOI | 0.959 |
| NAA | Thalamus | CPZ dose | 0.520 |
|  |  |  |  |

**Table S5. Association between neuropsychological scores and NAA levels (relative to total creatine) in ACC and thalamus.**

1. **Association between neuropsychological scores and NAA levels in the NR group**

|  | ACC | | | Thalamus | | |
| --- | --- | --- | --- | --- | --- | --- |
|  | correlation coefficient | p-value | q-value* | correlation coefficient | p-value | q-value* |
| Attention/working memory | 0.083 | 0.354 | 0.447 | -0.215 | 0.818 | 0.818 |
| Executive function | 0.072 | 0.372 | 0.447 | -0.164 | 0.755 | 0.818 |
| **Ideational fluency** | **0.551** | **0.004** | **0.023** | 0.487 | 0.017 | 0.103 |
| **Processing speed** | **0.505** | **0.008** | **0.025** | 0.020 | 0.468 | 0.818 |
| Verbal learning and memory | 0.125 | 0.284 | 0.447 | 0.131 | 0.291 | 0.818 |
| Visual learning and memory | -0.175 | 0.788 | 0.788 | -0.151 | 0.737 | 0.818 |

* Multiple comparison correction for 6 tests was conducted

1. **Association between neuropsychological scores and NAA levels in the R group**

|  | ACC | | | Thalamus | | |
| --- | --- | --- | --- | --- | --- | --- |
|  | correlation coefficient | p-value | q-value* | correlation coefficient | p-value | q-value* |
| Attention/working memory | -0.303 | 0.745 | 0.987 | 0.086 | 0.413 | 0.984 |
| Executive function | -0.372 | 0.795 | 0.987 | -0.008 | 0.508 | 0.984 |
| Ideational fluency | -0.745 | 0.973 | 0.987 | -0.477 | 0.903 | 0.984 |
| Processing speed | -0.511 | 0.880 | 0.987 | -0.711 | 0.984 | 0.984 |
| Verbal learning and memory | -0.624 | 0.933 | 0.987 | -0.314 | 0.795 | 0.984 |
| Visual learning and memory | -0.815 | 0.987 | 0.987 | -0.605 | 0.958 | 0.984 |

* Multiple comparison correction for 6 tests was conducted
